# The role of motivation in eating disorders: understanding sex differences in the circuits

**DOI:** 10.3389/fnbeh.2025.1644383

**Published:** 2025-09-08

**Authors:** Sofia Nasini, Antonino Casile, Brigitta Bonaldo, Camilla Mancini, Serafina Manila Guzzo, Luca Botticelli, Stefano Comai

**Affiliations:** ^1^Department of Pharmaceutical and Pharmacological Sciences, University of Padua, Padua, Italy; ^2^Pharmacology Unit, School of Pharmacy, University of Camerino, Camerino, Italy; ^3^Neuroscience Institute Cavalieri Ottolenghi (NICO), Turin, Italy; ^4^Department of Neuroscience “Rita Levi-Montalcini”, University of Turin, Turin, Italy; ^5^Department of Biosciences, Università degli Studi di Milano, Milan, Italy; ^6^Department of Psychology, Sapienza University, Rome, Italy; ^7^Fondazione Santa Lucia IRCCS, Rome, Italy; ^8^Department of Biomedical Sciences, University of Padua, Padua, Italy; ^9^Department of Psychiatry, McGill University, Montreal, QC, Canada

**Keywords:** eating disorders, sex differences, motivated behavior, neurotransmitters, neuronal pathways, murine models

## Abstract

Motivated behaviors, such as reproduction and feeding, are essential for mammalian survival. Although these behaviors serve distinct evolutionary purposes, they share a common function: fulfilling specific biological needs. Their regulation involves distinct brain regions and is influenced by a complex interplay of neural circuits, with significant sex-based differences. Alterations in motivation represent critical components of effort-based decision-making processes in eating disorders (EDs). Importantly, the impairments in motivated behavior observed in EDs arise not from structural changes within the relevant brain regions but rather from functional alterations influenced primarily by gonadal hormones. These hormones play a pivotal role in the pathophysiology of EDs, driving sex-based differences in both the qualitative aspects of symptom presentation and developmental trajectories through intracellular genomic signaling pathways. The current review examines sex differences in motivated behavior within the context of EDs.

## Highlights

- There are sex-based differences in how motivational processes are controlled and altered.
- In EDs, impaired motivated behavior is linked to brain activity rather than structural changes, and gonadal hormones play a significant role in this process.
- Gonadal hormones affect the pathophysiology of EDs and contribute to sex-based differences through intracellular genomic signaling.

## Introduction

Survival, reproduction, and feeding are fundamental behaviors driven by motivational processes essential for maintaining biological homeostasis and ensuring survival at both individual and species levels. Although these behaviors serve different evolutionary purposes, they share a common underlying neurobiological mechanism related to motivation (Salamone et al., 2016; Simpson and Balsam, 2016). As with most behaviors, motivation exhibits sex-based differences influenced by genetic, hormonal, and environmental factors (Kundakovic and Tickerhoof, 2024; Li et al., 2024; Massa and Correa, 2020). These differences do not arise from structural variations in brain regions or general neural circuitry, but rather from functional differences regulated primarily by gonadal hormones. These hormones exert their influence through both organizational (permanent effects occurring early in development) and activational mechanisms (temporary changes throughout life), shaping neural circuit activity rather than anatomical differences (Becker and Chartoff, 2019; Lenz et al., 2012). Consequently, genetic or environmental modifications can serve as risk factors that alter motivated behaviors (Kundakovic and Tickerhoof, 2024).

Eating Disorders (EDs), including anorexia nervosa (AN), bulimia nervosa (BN), and binge eating disorder (BED), are complex mental health conditions characterized by dysfunctional eating behaviors aimed at controlling body weight or coping with negative emotional states (American Psychiatric Association [APA], 2013).

Eating behavior is a motivated behavior that is significantly regulated by gonadal hormones and displays notable sex differences, potentially underlying the observed disparity in EDs prevalence between males and females. Specifically, disruptions in hormonal regulation alter the functional dynamics of motivational neural circuits, particularly by modulating dopaminergic and serotonergic signaling, synaptic plasticity, and the excitatory/inhibitory balance within key regions such as the prefrontal cortex (PFC), nucleus accumbens (NAc), and hypothalamus. These functional changes, rather than structural anomalies, contribute to the altered rewards processing, impulsivity, and emotional dysregulation commonly observed in individuals with EDs.

## Aim

This review aims to elucidate the role of sex-based differences in motivated behavior, emphasizing the increased vulnerability observed in females regarding alterations in motivation associated with EDs. Motivation is a multifaceted construct that encompasses several interrelated components, such as rewards sensitivity, emotional processing, cognitive control, and learning, which are regulated by partially overlapping neural circuits. Understanding how these interconnected systems are differentially modulated in males and females is crucial to clarify sex-related vulnerabilities in EDs. We particularly address how gonadal hormones regulate and influence the different behavioral components shaping motivation. These hormones exert both organizational (permanent structural and functional brain changes during critical developmental windows) and activational effects (temporary, hormone-dependent functional changes across the lifespan), thus contributing to the sex-based differences observed in the neural circuits underlying motivated behaviors. To support these considerations, we also refer to findings from preclinical models that have helped delineate the neurobiological substrates of sex-dependent motivational alterations relevant to EDs.

### Motivational processes and eating disorders: linking neurobiology to behavior

Understanding how motivated behaviors become dysfunctional is essential for elucidating the pathophysiology of psychiatric disorders, including EDs. Motivation refers to goal-directed behaviors aimed at achieving rewarding outcomes that satisfy essential needs. Although eating is vital for survival, its disruption may indicate underlying neurobiological alterations.

The mesocorticolimbic system, which includes the ventral tegmental area (VTA), NAc, limbic regions, and PFC, plays a central role in rewards processing and motivation (Kelley and Berridge, 2002; Robbins and Everitt, 2007). Within this circuitry, dopamine (DA) modulates rewards anticipation, reinforcement learning, and incentive salience (Eck and Bangasser, 2020). DA release in the NAc supports goal-directed actions, while DA activity in the striatum is associated with rewards consumption (Baldo et al., 2013; Small et al., 2003).

Compulsive and maladaptive behaviors, such as those seen in EDs, are linked to dysregulated DA signaling, particularly between the dorsolateral PFC and NAc (Ampel et al., 2016). Impaired self-control and altered rewards processing may thus contribute to pathological eating behaviors, reinforcing the relevance of dopaminergic circuits in EDs vulnerability (Leigh and Morris, 2018; Volkow et al., 2013).

### Motivated behavior as a key component in EDs

**FIGURE 1:**
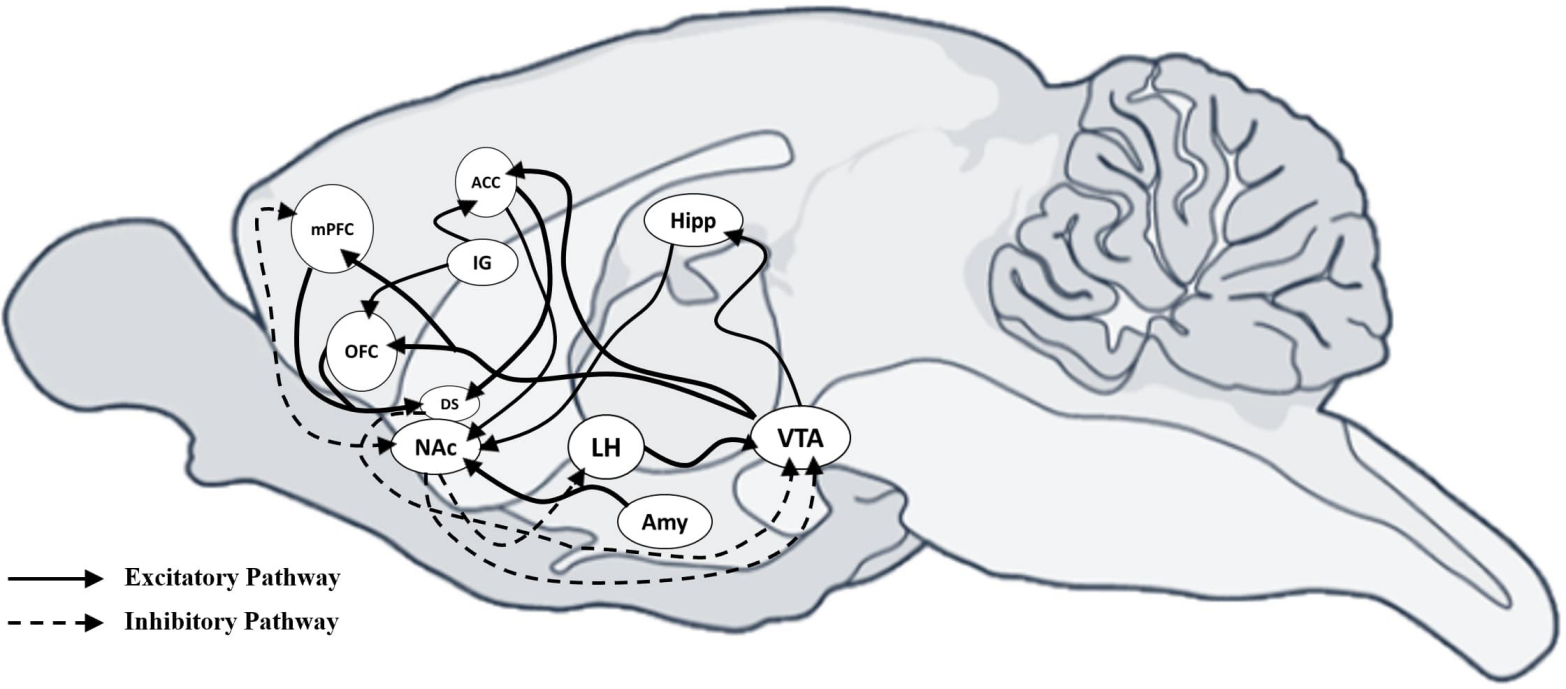
Sagittal view of mouse brain with excitatory and inhibitory connections between neural areas. Sagittal view of the mouse brain showing the excitatory and inhibitory connections between neural areas. Schematic representation shows the excitatory and inhibitory connections among different areas that drive motivational behaviors. Inhibitory projections are indicated with a dotted line, while excitatory projections are indicated with a solid line. mPFC, medial Prefrontal cortex; OFC, orbitofrontal cortex; ACC, anterior cingulate cortex; IG, indusium griseum; DS, dorsal striatum; NAc, accumbens nucleus; LH, lateral hypothalamic area; AMY, amygdala; Hipp, hippocampus; VTA, ventral tegmental area.

Eating is a motivated behavior essential for survival (Blundell and Rogers, 1991). According to the homeostatic feedback theory, food intake is regulated by physiological and behavioral mechanisms involving motivational systems. Cognitive processes, including hunger and satiety, are controlled by peripheral signals (e.g., ghrelin, leptin, insulin, cortisol) informing the central nervous system (CNS) about the body’s energy status. These signals trigger behaviors designed to regulate food-seeking and intake (Cosmides and Tooby, 2013; Deckers, 2018). Key brain regions involved, such as the PFC, orbitofrontal cortex (OFC), and anterior cingulate cortex (ACC), manage inhibitory and excitatory balance, crucial for processing rewarding stimuli and regulating emotional responses, especially in BN (Ikemoto, 2010; Kelley and Berridge, 2002; Robbins and Everitt, 2007; Figure 1).

Neuroplastic changes within these regions, influenced by both genetic predispositions and environmental factors such as stress, contribute to the development and maintenance of disordered eating behaviors (Bonaldo et al., 2024; Brewerton, 2015; Micioni Di Bonaventura et al., 2014). The brain’s rewards system comprises ventral and dorsal circuits: the ventral limbic system, including the anterior insula, ventral striatum, amygdala, OFC, and ACC, is responsible for recognizing rewarding stimuli and emotional responses, while the dorsal circuit, involving dorsal striatum, parietal cortex, and DLPFC, manages planning, regulation, and selective attention (Phillips et al., 2003). Dysfunction in these circuits, particularly in rewards processing and food intake regulation, contributes to maladaptive behaviors observed in EDs, such as BED and restrictive eating (Lee et al., 2017; Marazziti and Catena Dell’osso, 2008; Wang et al., 2023).

Clinically, AN, BN, and BED suggest dysregulation of motivational and rewards systems. In AN, reduced gratification from food restriction, and in BED, excessive food consumption to mitigate negative emotions, significantly contributes to the initiation and maintenance of these disorders (Monteleone et al., 2018). Such behaviors reflect underlying alterations in cognitive and motivational circuits regulated by neurotransmitters like DA, serotonin (5-HT), and neuropeptides (Bromberg-Martin et al., 2010; Johnson and Kenny, 2010).

Research indicates motivational changes in EDs result from intrinsic biological processes and environmental influences. For instance, a key symptom in EDs is anhedonia, a diminished ability to experience gratification linked to alter DA system. Food restriction, typical of AN, sensitizes DA pathways, whereas excessive food consumption in BED desensitizes these pathways, due to decreased DA receptor (DR) expression (Carr et al., 2003). These DA system alterations affect cognitive and motivational circuits, modulated by peripheral signals like leptin, ghrelin, glutamate, and opioids (Carr et al., 2010; Koizumi et al., 2009; Opland et al., 2010; Perello et al., 2010).

In AN, caloric restriction elevates DA release, promoting excessive physical activity as reward-seeking behavior. This heightened activity reinforces restrictive behaviors through 5-HT-mediated satiety signaling (Kaye et al., 2009; Keating et al., 2012). Conversely, recurrent binge-eating episodes in BED show neurobiological parallels with substance addiction, featuring functional abnormalities in neurotransmitter systems (DA, opioids) and impaired frontostriatal circuitry, underpinning impulsiveness and rewards craving (Novelle and Dieguez, 2018).

Therefore, eating behavior intricately involves motivational pathways modulated by genetic, hormonal, and environmental factors. Understanding these dysregulated motivational circuits provides insight into the neurobiological and psychological underpinnings of EDs.

These two circuits work together to evaluate environmental stimuli, associate them with rewards, and assess future consequences (Goldstein and Volkow, 2011).

Disruptions in these circuits, particularly in rewards processing and regulation of food intake, can lead to maladaptive behaviors such as binge eating or restrictive behaviors characteristic of EDs (Monteleone et al., 2018).

The clinical features of AN, BN, and BED suggest that EDs may stem from dysregulation of the motivational or rewards systems. The diminished sense of gratification from food restriction, and the excessive consumption of food to alleviate negative emotions observed in AN and BED, respectively, both contribute to the initiation and maintenance of harmful behaviors, which are closely related to alterations in cognitive functions that regulate eating behavior (de Souza et al., 2018).

Further research has indicated that these motivational changes are not solely due to external factors but may also be influenced by intrinsic biological processes, including altered signaling of key neurotransmitters.

**FIGURE 2:**
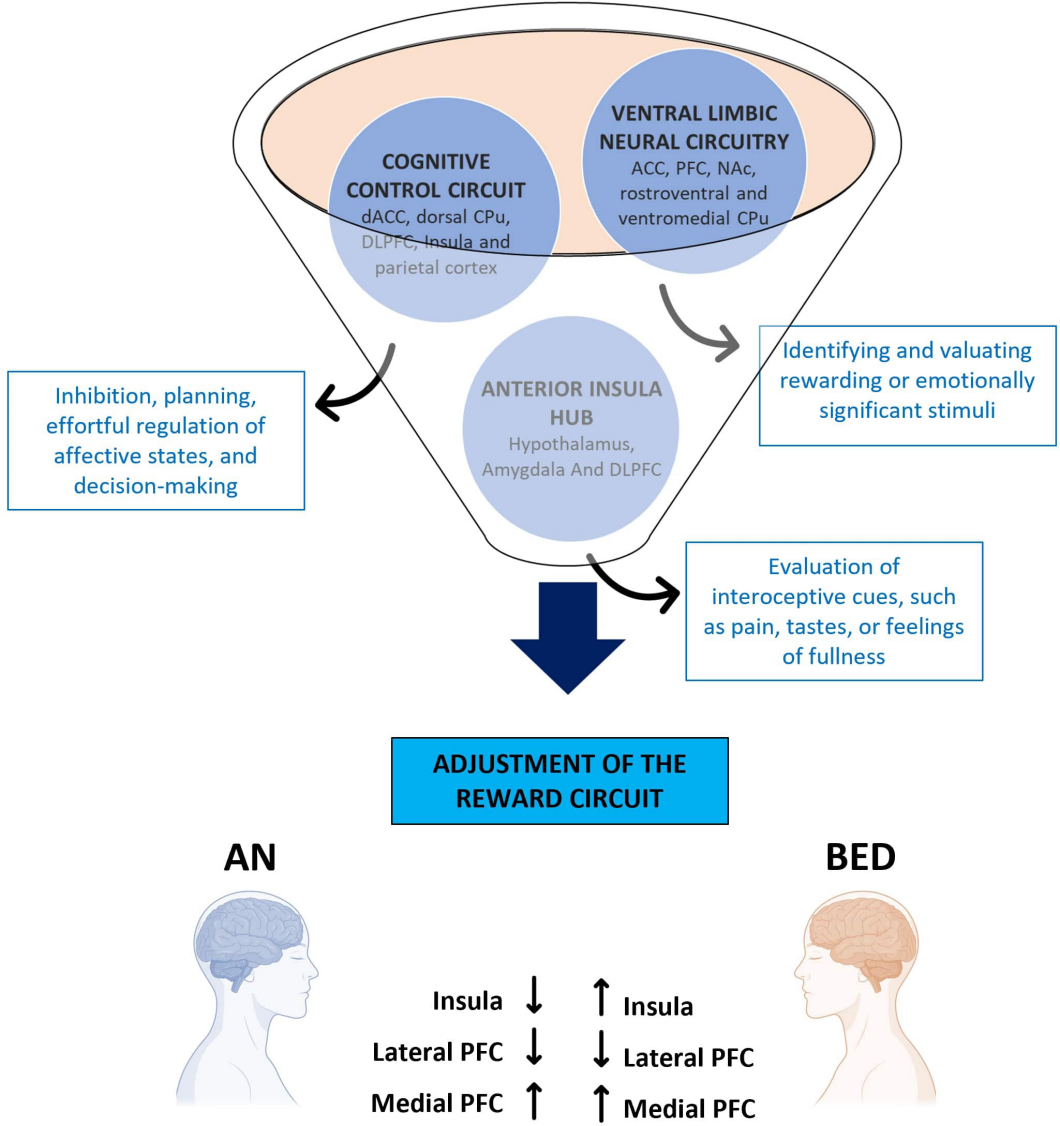
Brain areas involved in eating disorders. The representative diagram illustrates the different areas involved in the motivational circuitry, highlighting their relative functions and how they are differently altered in Anorexia Nervosa and Binge Eating Disorder. ACC, anterior cingulate cortex; AN, anorexia nervosa; BED, binge eating disorder; CPu, caudate putamen; dACC, dorsal anterior cingulate cortex; DLPFC, dorsolateral prefrontal cortex; NAc, nucleus accumbens; PFC, prefrontal cortex.

A key symptom in EDs is anhedonia, which is the reduced ability to experience gratification. This alteration impacts one of the primary rewards circuits, specifically the VTA. When rewarding stimuli are perceived, DA is released from the VTA, triggering a cascade of brain responses: it stimulates the NAc, promotes associative learning via the hippocampus, and processes emotions through the amygdala. The integration of these responses facilitates behaviors related to reward-seeking and learning. Research has shown that individuals with EDs exhibit altered DA systems, leading to changes in cognitive functions associated with rewards and motivation (Monteleone et al., 2018; Figure 2).

Food restriction, common in AN, has been linked to increased sensitization of DA pathways in rewards circuits (Carr et al., 2003). Conversely, the excessive food consumption characteristic of BED leads to desensitization of these circuits, caused by reduced expression of DRs (Johnson and Kenny, 2010). These changes in body weight are associated with impairments in both cognitive and motivational circuits, affecting DA signaling pathways. Moreover, altered production of leptin (Opland et al., 2010), ghrelin (Perello et al., 2010), glutamate (Carr et al., 2010), and opioids (Koizumi et al., 2009) further disrupt the rewards system.

Increased body weight, particularly in the case of BED, has been shown to negatively impact the regulation of DRs sensitivity, leading to dysregulated feeding behaviors.

Changes in body weight also correlate with impairments in neural circuits involved in motivation. In AN, alterations in neuropeptides related to appetite, the hypothalamic-pituitary-adrenal (HPA) axis, 5-HT, and DA pathways are observed (Kaye, 2008; Sodersten et al., 2008). Caloric restriction in AN triggers an increase in DA release, which in turn promotes exercise as a reward-seeking behavior. This increased motor activity provides a sense of fulfillment through the release of 5-HT, which in turn promotes satiety and reduces food intake (Kaye et al., 2009). The excessive release of neurotransmitters in AN alters motivational circuits and, as a result, emotional perception and the sense of fulfillment (Keating et al., 2012).

Recurrent binge eating episodes in BED share similarities with behaviors seen in substance dependence (Novelle and Dieguez, 2018). Functional abnormalities in neurotransmitter systems (e.g., DA and opioids) and frontostriatal changes are key characteristics of BED, such as loss of control over impulsiveness and the craving for rewarding stimuli. These symptoms reflect underlying alterations in motivational circuits (Novelle and Dieguez, 2018).

As with other addictive behaviors, the loss of control in BED may be attributed to alterations in the reinforcement learning pathways, which are critical in driving behaviors related to food intake and emotional regulation (Monteleone et al., 2018).

In conclusion, eating behavior is intricately linked to motivation and regulated by both peripheral and central signaling pathways. EDs disrupt these pathways, leading to significant alterations in rewards systems. These disruptions affect how individuals with EDs perceive and process emotional states, rewards, and gratification (Monteleone et al., 2018).

Given the role of motivation in eating behavior, it follows that disruptions in motivational circuits are central to EDs pathology. Understanding how these circuits become dysregulated can provide insights into both the neurobiological and psychological aspects of EDs.

#### Biological sex

It plays a key role in shaping motivational processes and vulnerability to EDs, primarily through hormonal and neurobiological mechanisms. Estrogen (E) and testosterone (T) influence rewards sensitivity and food intake by modulating mesolimbic circuits and emotional regulation pathways (Eck and Bangasser, 2020). In females, heightened dopaminergic reactivity and greater activation of emotional networks, especially during hormonal fluctuations, can amplify responses to both food-related and affective stimuli (Eck and Bangasser, 2020). These findings highlight the importance of incorporating sex-specific factors into EDs research and treatment strategies (Asarian and Geary, 2006; Castellini et al., 2016).

### Sex differences in neural circuits underlying motivation

As previously discussed, motivation and other behaviors exhibit sex differences that depend on genetic predispositions, hormonal influences, and environmental contexts. Gonadal hormones are particularly critical, exerting both organizational effects, permanent alterations occurring early in development, and activational effects, which are temporary and hormone-dependent changes throughout life (Arnold and Breedlove, 1985; Blencowe et al., 2022).

Importantly, these hormonal effects influence functional neural activity rather than gross anatomical structures, shaping sex differences in brain regions implicated in motivation.

Despite complexity in the underlying mechanisms, sex-related regions involved in emotional and cognitive processing contribute substantially to motivated behaviors (Eck and Bangasser, 2020). To elucidate how sex differences shape motivation, we examine the anatomical organization of the neural circuits underlying reward-related behaviors, with particular attention to sex-specific features and hormonal influences.

#### Sexual differences in motivated behavior in EDs

While there is growing recognition and diagnosis of EDs in men, they remain significantly more prevalent in women (Garcia et al., 2020; Micioni Di Bonaventura et al., 2020; Murray et al., 2017), a disparity largely rooted in sex-specific neuroendocrine mechanisms that modulate motivation and rewards processing.

Steroid hormones modulate CNS function and behavior mainly via intracellular genomic signaling, as evidenced by animal and human studies. The organization of the CNS is profoundly shaped by hormonal signals starting from prenatal development and continuing through puberty, with responsiveness to these hormones further modified by activational effects during adolescence and adulthood (Arnold, 2009; Moraga-Amaro et al., 2018; Schulz and Sisk, 2016). Consequently, both organizational and activational effects of sex steroids fluctuate dynamically across life stages.

Regarding activational influences of ovarian hormones, E exerts direct anorexic effects, whereas progesterone promotes food intake by antagonizing the effects of E (Asarian and Geary, 2006). Physiological fluctuations in ovarian hormones, as observed in animal models during estrous cycles and in women across menstrual cycles, correlate with alterations in eating behaviors, such as BED and AN. For instance, female rats exhibit larger binge episodes during diestrus or proestrus, with lower levels during estrus (Alboni et al., 2017; Micioni Di Bonaventura et al., 2017).

Abnormalities in rewards and punishment sensitivity, modulated by hormonal and environmental factors, may further elevate vulnerability to binge eating and purging behaviors. Heightened rewards sensitivity potentially increases the likelihood of binge eating, while increased sensitivity to punishment may enhance compensatory behaviors. For example, women with the binge/purge subtype of AN exhibit significantly elevated rewards sensitivity (Harrison et al., 2010), whereas women with BN display a strong correlation between rewards sensitivity and purging frequency (Farmer et al., 2001). It has been proposed that purging behaviors decrease brain acetylcholine (ACh) levels, reducing associated negative sensations.

Neuroimaging studies further demonstrate that women with BN show significantly greater activation of rewards pathways when viewing food images compared to healthy controls (Brooks et al., 2011). Animal models similarly suggest females have a heightened preference for palatable food, linked to increased activation in mesolimbic rewards circuits (Sinclair et al., 2017). Post-pubertal female rats also show stronger preferences for sweet tastes compared to males, influenced by circulating *E*s and T exposure (Wade and Zucker, 1969).

T modulates rewards sensitivity through interactions with the DA system. Prenatal T exposure in humans correlates with increased rewards sensitivity and higher impulsivity in females (Lombardo et al., 2012). Elevated perinatal T exposure affects the dopaminergic system differently in males and females. In male rodents, there is higher density of DA D1 receptors in the NAc during the perinatal phase, similar to pubertal females. T can also alter nigrostriatal responses to DA by binding to ARs, influencing gene expression of DA transporters and DAD2 and D3 receptors in the substantia nigra (SN) and striatum.

In contrast, *E* modulates neural responsiveness in brain regions responsible for affective processing and eating behavior, including the amygdala, PFC, NAc, paraventricular nucleus (PVN), and the bed nucleus of the stria terminalis (BNST). These areas regulate motivated behaviors through mesocorticolimbic DA pathways. E2 indirectly affects dopaminergic systems via cholecystokinin (CCK), a neuropeptide critical for satiation signaling. DA neurons projecting from the VTA to medial posterior NAc co-release CCK, enhancing signaling and decreasing food intake during ovulatory or estrous phases (Vaccarino, 1994). Simultaneously, CCK induces DA release within rostral and caudal NAc regions, modulating cAMP activation. Female rodents exhibit greater flexibility in mesolimbic DA transmission, with E2 likely mediating adaptive changes in motivated behaviors. *E* and DA activities demonstrate an inverted-U relationship regarding food rewards behaviors, with suppression at peak *E* levels, possibly mediated via E2 receptors stimulating 5-HT neurons in dorsal raphe nucleus, thus reducing binge-like eating behaviors, particularly involving fats (Cao et al., 2014). AN is linked to heightened responsiveness in brain rewards circuits, potentially due to hypersensitive DA systems. Although the precise mechanisms by which *E* modulates DA systems to reduce palatable food consumption remain unclear, striatal D1 expression is higher in males compared to females, whereas E2 rapidly reduces D2 binding in females, suggesting significant modulation of DA systems by *E* specifically in females (Becker, 1990, 2005).

*E*s also indirectly influence DA activity in mesolimbic pathways by modulating glutamatergic and GABAergic neurotransmission. Abnormal glutamatergic signaling has been observed in medium spiny neurons (MSNs) of the NAc in human EDs patients (Keating et al., 2012; Wierenga et al., 2015; Zastrow et al., 2009). *E*s enhances glutamate transmission while suppressing GABAergic transmission, contributing to disruptions seen in EDs.

This interplay forms a neurocircuit involving glutamatergic neurons in the PFC, GABA interneurons, and DA neurons in the VTA and SN, balancing DA activity and reward-related behaviors. Increased GABAergic inhibition of DA neurons in EDs contexts can elevate firing rates, causing reward-related bursts (Paladini and Roeper, 2014).

Lastly, orexin (ORX) neuropeptides are implicated in EDs, particularly in females. Hypothalamic ORX neurons projecting to DA, 5-HT, and GABA/glutamate brain areas enhance the drive for palatable foods, significantly influencing binge-like and anorectic behaviors, especially in female rodents. Pharmacological blockade of ORX reduces motivation for palatable food in both sexes (Freeman et al., 2021).

Overall, maturation of these systems during puberty, influenced by Es and T, may heighten susceptibility to EDs during this developmental stage (Friemel et al., 2010; Iughetti et al., 2011). Investigating sex differences in these neural mechanisms provides essential insights for targeted treatments for EDs (Klump, 2013).

#### Sex differences in the mesocorticolimbic rewards system

Key brain regions involved in motivation, rewards, and emotional regulation exhibit sex-specific characteristics that may explain the greater vulnerability to EDs observed in females (Culbert et al., 2016, 2018, 2021). These differences are primarily shaped by the organizational and activational effects of gonadal hormones on dopaminergic and GABAergic signaling (Arnold, 2009; Arnold and Breedlove, 1985).

In the VTA, females show a higher density of dopaminergic neurons (Rincon-Cortes and Grace, 2017) and increased DA release during the estrous phase, modulated by E2 (Zhang et al., 2008). This contributes to greater rewards sensitivity and cycle-dependent fluctuations in food motivation (Zhang et al., 2008). Androgens also influence VTA function, although their mechanisms are less well characterized (Sato et al., 2008; Shughrue et al., 1997).

The striatum, involved in motor control and reinforcement learning, exhibits sex-dependent functional modulation through E2 (Gerfen and Surmeier, 2011; Yager et al., 2015). While anatomical differences are minimal, E2 enhances DA transmission and adjusts GABAergic activity, contributing to behavioral regulation and inhibitory control, particularly relevant in compulsive eating (Gerfen and Surmeier, 2011; Yager et al., 2015).

The NAc, a key hub for integrating rewards and motivation, shows structural and functional differences between sexes (Becker and Hu, 2008; Yoest et al., 2014). In females, MSNs are more excitable, and DA release varies with hormonal cycles, potentially driving stronger responses to palatable food and greater vulnerability to binge episodes (Dorris et al., 2015; Wissman et al., 2011).

In the substantia nigra (SN), testosterone increases GABAergic neuron density, while E2 enhances DA function, reflecting sex-specific receptor expression (Rincon-Cortes and Grace, 2017; Sarvari et al., 2014). These adaptations affect the broader rewards circuitry and motivated behaviors (Kritzer, 1997; Wilson, 1993).

### Sex differences in emotional processing and motivated behavior

In addition to the mesocorticolimbic rewards circuitry described above, motivated behaviors are strongly influenced by emotional processes. Emotional regulation involves key limbic areas such as BNST and the amygdala. These regions show marked sex-based differences in animal models, which could help explain the observed disparities between males and females in susceptibility to EDs.

#### BNST

Animal studies indicated that the BNST plays a crucial role in the regulation of anxiety, stress, and motivated behaviors, including food intake (Ortiz-Juza et al., 2021). In rodents, particularly female rats, the BNST significantly contributes to binge eating episodes, influenced by environmental stressors such as early-life adversity or chronic stress exposure (Micioni Di Bonaventura et al., 2014, 2017). While male rodents exhibit a larger overall BNST volume, female rodents show enhanced sensitivity to hormonal modulation within this region, especially to E2. Elevated E2 levels in female rats upregulate glutamatergic neuronal activity, directly influencing emotional and feeding behaviors (Herbison and Fenelon, 1995; Morgan et al., 2004). Furthermore, neuropeptides such as OXT and vasopressin also modulate BNST activity differently in males and females, contributing to sex-dependent responses in stress-related, social, anxiety-related, and feeding behaviors (Bonaldo et al., 2021; de Vries, 2008; Graic et al., 2018).

Similarly, the amygdala, which processes emotional stimuli, also exhibits sex-based differences in the regulation of motivated behavior.

#### Amygdala

The amygdala is a complex structure involved in processing emotional (Gallagher and Chiba, 1996), fearful (Andero et al., 2016), or rewarding stimuli and, similarly to the BNST, plays a crucial role in non-homeostatic food-related behaviors, particularly binge eating episodes observed in females (Bohon and Stice, 2012; Pringle et al., 2011) and female rats (Blasio et al., 2013; Micioni Di Bonaventura et al., 2019).

Its functioning is significantly influenced by environmental experiences, such as chronic stress or societal pressures regarding body image, which further modulate susceptibility to BED in people. The amygdala receives extensive inputs from cortical regions, thalamus, hippocampus, and olfactory bulb, and projects to limbic, cortical, and midbrain regions, regulating the production of neurotransmitters such as norepinephrine (NA), 5-HT, Ach, and DA (Takenawa et al., 2023).

Structurally, the amygdala comprises the centromedial (CeA and MeD), basolateral (BLA), and cortical (CO) subregions (Knapska et al., 2007). These subnuclei finely regulate emotional perception and processing, displaying clear morphofunctional sex differences (Casile et al., 2024; Knapska et al., 2007). For instance, female rodents exhibit increased dendritic spine density, a phenomenon modulated by E levels sensitive to environmental factors, such as stress exposure, and heightened during estrous and proestrus phases (Blume et al., 2017; Calandreau et al., 2005).

Furthermore, amygdala subregions display distinct populations of GABAergic and glutamatergic neurons, establishing a region-specific excitatory-inhibitory balance tightly regulated by circulating E2 levels (Blume et al., 2017; Dalpian et al., 2019; Martinez et al., 2006).

These hormonal fluctuations impact neurotransmission both within the amygdala and across interconnected circuits, emphasizing the intricate interplay between gonadal hormones, environmental contexts, and emotional behavior regulation (Calandreau et al., 2005).

**FIGURE 3:**
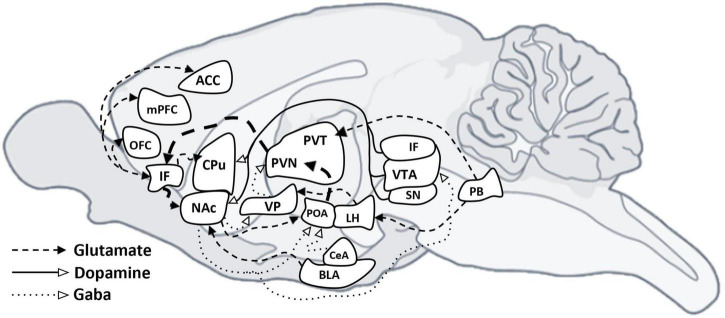
Neural circuits underlying motivated “desire” and hedonic “satisfaction.” Diagram summarizing cell connections between limbic, cortical and midbrain nuclei. GABAergic projections are indicated with a dotted line, dopaminergic projections are indicated with a solid line, and glutamatergic projections are indicated with a dashed line. mPFC, medial prefrontal cortex; OFC, orbitofrontal cortex; ACC, anterior cingulate cortex; IF, interfascicular nucleus; NAc, accumbens nucleus; CPu, caudate putamen (striatum); VP, ventral pallidum; PVN, paraventricular hypothalamus nucleus; PVT, paraventricular thalamic nucleus; POA, medial preoptic area; LH, lateral hypothalamic area; BLA, basolateral amygdaloid nucleus, anterior part; CeA, central amygdaloid nucleus, anterior part; SN, substantia nigra; VTA, ventral tegmental area; PB, parabrachial nucleus.

Overall, this evidence underlines significant sex differences in brain regions modulating emotional and motivational behaviors. These dimorphisms, evident at anatomical, neurochemical, and functional levels, highlight how gonadal hormones interact dynamically with environmental influences, contributing to differential susceptibility between sexes to maladaptive eating behaviors characteristic of EDs (Agoglia et al., 2020; Johnson et al., 2021), which will be explored in greater depth in subsequent sections (Figure 3).

## Conclusion

Eating disorders, including AN, BN and BED, are complex psychiatric conditions characterized by dysregulated eating behaviors, such as abnormal weight control, extreme dietary restrictions, binge eating, compensatory behaviors, and pathological concerns with body image. These behaviors are frequently accompanied by psychological traits such as low self-esteem, perfectionism, emotional instability, and social withdrawal. Comorbid conditions such as anxiety and depression are also common and further exacerbate ED symptomatology, significantly impairing quality of life.

Eating, a motivated behavior essential for survival and homeostasis; however, under certain pathological conditions, the neurobiological regulation of eating becomes disrupted, contributing to ED pathogenesis. Central to motivated behavior and rewards processing is the mesocorticolimbic system, which integrates emotional, cognitive, and reward-related information. As highlighted in this review, this circuitry exhibits pronaunced sex differences due to gonadal hormones through both organizational and activational mechanisms. Preclinical studies demonstrate that sex hormones modulate DA neurotransmission in the mesocorticolimbic system and also can indirectly influence GABAergic and glutamatergic activity. These sex-dependent influences may help explain differential vulnerability to EDs between males and females. Nonetheless, the neurobiological basis of these sex differences remains insufficiently understood and warrants further investigation, particularly into how hormonal modulation affects motivated behavior and its dysregulation in EDs.

### Clinical and translational implications

In humans, EDs emerge from a multifactorial interplay between biological predispositions and non-biological influences. Sociocultural norms, environmental exposures, and psychosocial stressors critically shape the onset, trajectory, and clinical expression of these complex conditions. In parallel, epigenetic mechanisms, responsive to early-life experiences, nutritional status, and chronic stress, may induce persistent changes in gene expression, thereby influencing neural circuits involved in motivation, emotion regulation, and rewards processing. Importantly, these environmental and epigenetic factors may interact with sex-specific hormonal and neurobiological substrates, ultimately contributing to individual variability in symptom presentation, disease severity, and treatment response. To advance our understanding of EDs pathophysiology, it is critical to bridge preclinical insights with human studies. Integrating findings from animal models with neuroimaging, genetic, and epigenetic research in clinical populations could accelerate the identification of novel biomarkers and therapeutic targets, while increasing our knowledge of the underlying neurobiology. Recognizing sex as a fundamental biological variable in both basic and translational research is essential for the development of personalized and more effective interventions. Future research should prioritize longitudinal and interdisciplinary approaches that account for sex differences, hormonal status, and environmental exposures to better elucidate the complex and dynamic neurobiology underlying EDs.
